# Efficacy of B-TACE Versus C-TACE and Potential Predictive Value of Intraoperative Balloon-Occluded Stump Pressure in HCC

**DOI:** 10.3390/jcm15020668

**Published:** 2026-01-14

**Authors:** Liting Shan, Zhuoyang Fan, Guowei Yang, Sheng Qian, Wei Zhang, Bo Zhou, Rong Liu

**Affiliations:** 1 Shanghai Institute of Medical Imaging, Fudan University, Shanghai 200032, China; 2 Zhongshan Hospital, Fudan University, Shanghai 200032, China

**Keywords:** hepatocellular carcinoma, transarterial chemoembolization, balloon-assisted TACE, embolization pressure, predictive biomarker

## Abstract

**Objectives:** To compare the therapeutic efficacy and safety of balloon-assisted transarterial chemoembolization (B-TACE) versus conventional TACE (C-TACE) in hepatocellular carcinoma (HCC) and to evaluate the potential predictive value of intraoperative balloon-occluded arterial stump pressure (Boasp). **Methods:** In this prospective, single-centre, randomized controlled study, 60 patients with hepatocellular carcinoma were allocated to either the B-TACE group (*n* = 30) or the C-TACE group (*n* = 30). One patient in the B-TACE group was lost to follow-up after allocation. The primary analyses were conducted according to the intention-to-treat (ITT) principle, including all randomized patients, with conservative handling of missing data. Sensitivity analyses were performed to assess the robustness of the results. Tumor response and survival outcomes were evaluated using the modified Response Evaluation Criteria in Solid Tumors (mRECIST) and Cox proportional hazards regression models. Intraoperative balloon-occluded arterial stump pressure (BOASP) was measured as an exploratory parameter to quantify embolization adequacy. Adverse events (AEs) were systematically assessed and graded according to the Common Terminology Criteria for Adverse Events (CTCAE), version 5.0. **Results:** TACE achieved a higher 3-month ORR (63.3% vs. 10.0%, *p* < 0.001) and 6-month disease control rates (80.0% vs. 36.7%, *p* < 0.001), with PFS (HR = 0.30, 95% CI 0.148–0.608) and procedures within 6 months (1 vs. 3, *p* < 0.001). The 6-month surgical conversion rate was higher (34.5% vs. 6.7%, *p* = 0.009). Changes in Boasp correlated with efficacy (AUC = 0.825, *p* = 0.0398). Severe infections were lower in B-TACE (17.2% vs. 76.7%, *p* < 0.001). **Conclusions:** B-TACE offers superior efficacy, survival, and surgical conversion versus C-TACE with favorable safety. Boasp provides a quantitative biomarker for predicting treatment response.

## 1. Introduction

Hepatocellular carcinoma (HCC) is one of the most common malignancies worldwide, characterized by high mortality and incidence [1]. For patients who are ineligible for radical treatment with intermediate or advanced stage HCC, the major local therapeutic strategy is TACE [2]. However, C-TACE has several deficiencies such as the non-homogeneous distribution of drugs and incomplete embolization with high local recurrence rate [3]. As a result, there is an unmet need for improved therapies. Lately, with the improvement of interventional technology, balloon-assisted chemoembolization (B-TACE) has been gradually introduced into clinical practice [4].

The key to B-TACE is to redistribute blood flow to the target lesion, thereby allowing chemotherapy drugs/radionuclides to concentrate within the tumor, enabling increased drug residence time in the tumor area while preserving surrounding parenchyma, potentially improving local treatment effectiveness and prolonging patient survival [5,6,7]. Some small-sample studies have indicated that B-TACE may offer advantages over C-TACE regarding tumor response rates. However, high-quality prospective evidence regarding its long-term efficacy and underlying mechanisms remains insufficient [8,9].

In previous animal experiments conducted by our research group, we utilized a porous medium model of the renal artery/renal system in rabbits, employing Computational Fluid Dynamics (CFD). These experiments confirmed that changes in blood flow resistance within the terminal vessels during embolization were critical factors influencing local arterial hemodynamics. We developed a predictive equation that delineates the relationship between the injection of the embolic agent and hemodynamic parameters [10]. This equation facilitates the quantitative assessment of the degree of embolization through local blood pressure measurements, providing essential mechanical parameters for clinical translation. However, significant differences exist between the microenvironment of animal models and human tumors [11,12]. Our previous study demonstrated that when the arterial residual pressure (BOASP) at the tip of the inflated microcatheter was measured at 64 mmHg or lower, there was enhanced penetration of chemotherapy drugs and embolic materials into the tumor and its blood vessels. In contrast, when BOASP did not reach 64 mmHg, the concentration of lipidol emulsion and the proportion of embolized liver parenchyma in hepatocellular carcinoma (HCC) were significantly lower [5]. This complexity complicated the establishment of a reliable efficacy prediction model based solely on data from animal experiments [13]. Therefore, this study designed a prospective randomized controlled study to compare B-TACE with C-TACE in HCC patients and to explore BOASP, using pressure sensors during B-TACE to record when the balloon was not inflated (P0) without embolus injected intraoperatively and when the balloon unexpanded pressure (P0), expanded pressure (PI), expanded pressure (P2), and retracted pressure (P3) after balloon injection, and to predict prognosis by changes in BOASP (P2-P1/P0), in order to provide new evidence for broader clinical application and individualized treatment of B-TACE.

## 2. Material and Methods

### 2.1. Study Design

This prospective study was approved by the Institutional Review Board. The protocol was approved by the Medical Ethics Committee of Zhongshan Hospital affiliated to Fudan University and adhered to the principles outlined in the Declaration of Helsinki and China regulations.

### 2.2. Patient Characteristics

Inclusion criteria: 1. Age 18–80, any gender; 2. Diagnosed with hepatocellular carcinoma per specified guidelines; 3. BCLC tumor stage A–C; 4. Expected survival ≥ 3 months; 5. Untreated target lesions, diameter 30–200 mm; 6. Suitable blood and organ function for intervention. Exclusion criteria: 1. Recent local or TACE treatments; 2. Prior systemic anticancer therapy; 3. Recent thrombotic events; 4. Coagulation disorders; 5. Portal vein trunk occlusion; 6. High risk of untreatable TACE or embolism.

### 2.3. Randomization and Treatment Procedures

This study was designed as a prospective, randomized controlled study conducted at a single center. A total of 70 patients were enrolled from 15 April 2024 to 15 April 2025, 10 of whom were ineligible, and all eligible patients were randomly assigned (1:1) to the B-TACE or C-TACE group using a computer-generated random sequence. Randomization was performed by an independent investigator not involved in patient enrollment or outcome assessment. Allocation concealment was ensured using sealed, opaque envelopes. This study is a report of a randomized controlled trial conducted in accordance with the CONSORT statement (see Supplementary Materials Table S1).

Given the exploratory nature of this single-center pilot study, a formal sample size calculation was not performed. The sample size was determined based on feasibility and prior institutional experience.

**Figure 1:**
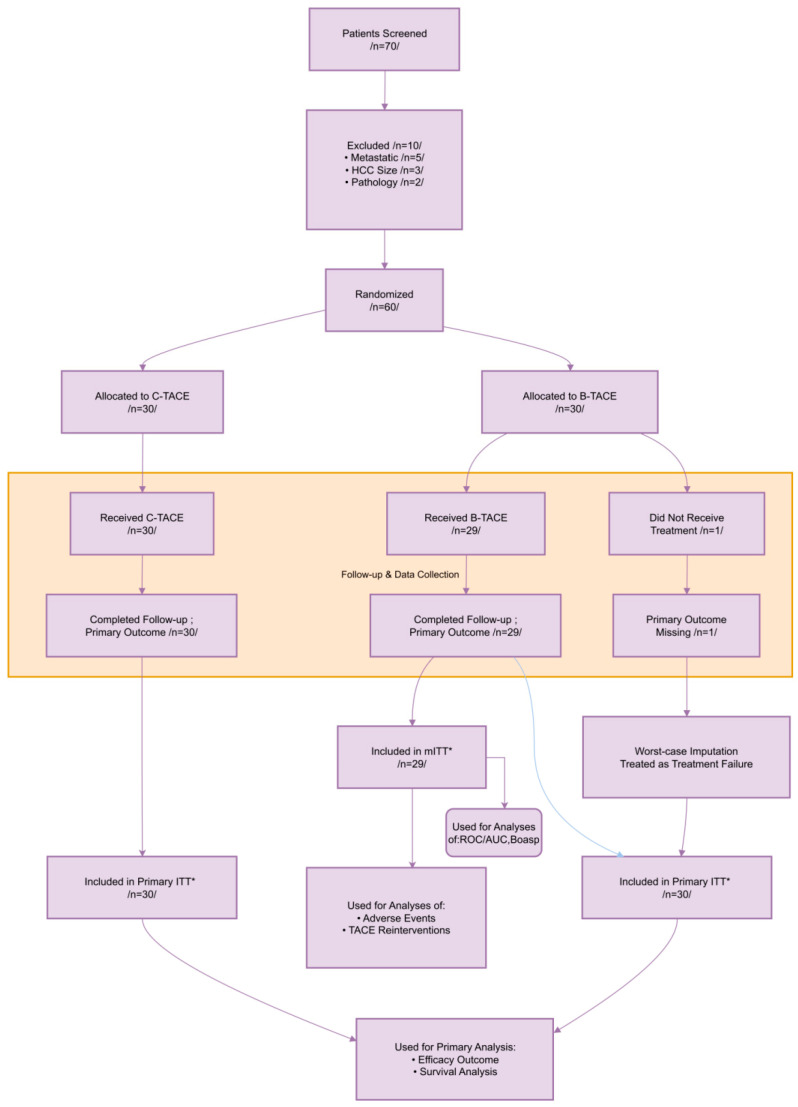
This diagram illustrates the flow of participants from screening to analysis. The primary efficacy analysis adhered to the intention-to-treat (ITT) principle, with a worst-case imputation applied to the one patient in the B-TACE group who did not receive the intervention (imputed as treatment failure). Some analyses (e.g., adverse events) were based on a modified ITT (mITT) population comprising patients who completed the intervention and follow-up. mITT: Included all patients who received the allocated intervention and completed follow-up. * Primary ITT: Included all randomized patients, with missing data handled by worst-case imputation.

All eligible patients were randomized within 1 day of eligibility confirmation and started treatment within the following 3 days. In the B-TACE group, one patient was unwilling to receive treatment due to personal reasons, so it was included as a deletion case. All surgeries were performed by a chief physician or associate chief physician in the study. Due to the nature of the interventional procedures, blinding of operators and patients was not feasible. All patients were evaluated for tumor location and characteristics preoperatively using digital subtraction angiography (DSA) and preoperative imaging (MRI) (Figure 1).

### 2.4. B-TACE and BOASP Measurement

**Figure A1:**
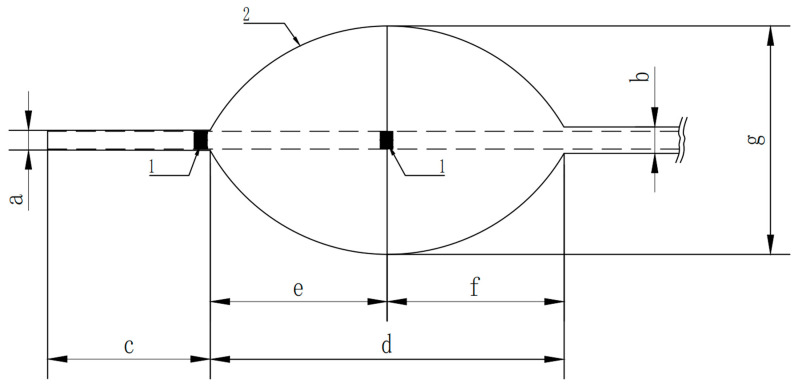
Catheter tip and microballoon schematic diagram (Huanxin Medical Clinical Supply Company, Suzhou, China) a = 1.9-French, b = 2.7-French d = balloon inflation length, c is the tip of the catheter to the balloon section, e = f = d/2 (mm) (mm, ±3 mm) g = balloon inflation diameter, 1 is radiopaque marker, 2 is balloon. BOASP measurement used 2.7 fr tip microballoon catheter.

The right common femoral artery was used as the approach. All patients were treated under local anesthesia with arterial puncture using an 18-gauge needle without incising the skin, and a 2.7 fr-tip microballoon catheter was inserted using a 5-Fr sheath guidewire. Details of the balloon microcatheter are provided in the Figure A1. Real-time full-range pressure monitoring during embolization is not possible since pressure measurement requires catheter access. BOASP was recorded by an extracorporeal pressure sensor connected to a microballoon catheter. 4 real-time intraoperative balloon-occluded stump pressure measurements were performed with a pressure transducer during the operation: balloon unexpanded pressure (P0), balloon expanded pressure (P1), balloon expanded pressure (P2), and balloon retracted pressure (P3) after balloon injection. Each measurement was repeated to ensure the stability of the signal, and the average value was analyzed. Under the condition of balloon dilation blocking, lipiodol and gelatin sponge particles or microspheres were injected to plug the balloon. After the plug was completed, the balloon was kept inflated until the pressure stabilized, and then the balloon was withdrawn. The choice of plugging agent was determined by the surgeon according to the patient’s condition. The microballoon catheter was advanced near the tumor feeding artery. DynaCT combined with DSA was used to evaluate tumor occlusion.

### 2.5. Endpoint Assessment

Due to the short follow-up period of this study, the primary endpoint was PFS, 6-month DCR, progression-free survival (PFS): the time from enrollment to tumor progression or death due to any cause (judged by the investigator according to mRECIST criteria). 6 months DCR: Defined as the proportion of patients who have achieved complete response (CR), partial response (PR), or stable disease (SD) due to treatment intervention. Secondary endpoints included objective response rate (ORR) at 4–8 weeks after B-TACE, proportion of patients defined as CR, PR, and tumor response at 6 months divided into CR, PR, SD, and PD. Tumor response assessment was performed according to enhanced CT or enhanced MRI assessment (mRECIST criteria). Venous non-enhancement areas were defined as necrotic areas, and all assessments were performed by an associate director and a director level physician. The last follow-up date was 1 October 2025.

### 2.6. Safety Assessment

Postoperative adverse events (AEs) were systematically recorded during follow-up and assessed according to standardized criteria. All adverse events were graded using the Common Terminology Criteria for Adverse Events (CTCAE), version 5.0. Only severe adverse events, defined as CTCAE grade ≥ 3.

### 2.7. Statistic

All statistical analyses were conducted using GraphPad Prism 10.0 (GraphPad Software, San Diego, CA, USA) and SPSS version 30.0. The primary endpoint was analyzed strictly according to the intention-to-treat (ITT) principle, including all randomized patients. One patient in the B-TACE group was lost to follow-up after allocation, and the primary outcome was conservatively handled in the ITT analysis. Sensitivity analyses using a worst-case imputation approach were performed to assess the robustness of the results. Secondary and exploratory analyses were conducted using modified ITT or complete-case populations, as specified for each analysis. For analyses requiring complete data, such as adverse events, BOASP-related analyses, ROC/AUC evaluation, and the number of TACE reinterventions within 6 months, patients without available data were not included in the corresponding analyses. Continuous variables were tested for normality and are presented as mean ± standard deviation (SD). Group comparisons were performed using independent-sample *t*-tests or the Mann–Whitney U test, as appropriate. Comparisons among more than two groups were conducted using one-way analysis of variance (ANOVA) or the Kruskal–Wallis test, with appropriate post hoc adjustments. Categorical variables were compared using the χ^2^ test or Fisher’s exact test when the expected frequency was <5. For comparisons of intraoperative BOASP values before (PT1) and after embolization (PT2) within the same patients, paired *t*-tests were used. Postoperative adverse events were systematically evaluated and graded according to the Common Terminology Criteria for Adverse Events (CTCAE), version 5.0.

The number of TACE reinterventions within 6 months was compared using the Mann–Whitney U test, and data distributions were visualized using box plots and scatter plots. Survival curves were generated using the Kaplan–Meier method and compared using the log-rank test. Hazard ratios (HRs) and 95% confidence intervals (CIs) were estimated using the Cox proportional hazards regression model. Receiver operating characteristic (ROC) curves and the area under the curve (AUC) were used to evaluate the potential predictive performance of exploratory efficacy indicators. Subgroup analyses were performed using stratified Cox regression, with HRs and 95% CIs presented in forest plots. All statistical tests were two-sided, and a *p* value < 0.05 was considered statistically significant.

## 3. Results

### 3.1. Baseline Characteristics

A total of 60 patients with hepatocellular carcinoma undergoing transarterial chemoembolization were enrolled in this study. Of these, 30 patients were randomly allocated to receive B-TACE, and 30 patients were allocated to receive C-TACE. Baseline characteristics were assessed in all randomized patients. Comparative analysis demonstrated that the two groups were well balanced with respect to most baseline parameters, with no significant differences in demographic or clinical characteristics between the groups. However, there were some interesting trends and one significant difference between groups. The proportion of patients with BCLC stage C (73.3% vs. 50.0%, *p* = 0.07) and the proportion of patients with single tumors (73.3% vs. 46.7%, *p* = 0.07) were higher in the B-TACE group, although these differences did not reach statistical significance. Crucially, the distribution of serum AFP levels was significantly unbalanced (*p* < 0.01). The proportion of patients with AFP *>* 200 ng/mL was significantly higher in B-TACE group (66.7%) than in C-TACE group (30.0%). Baseline demographic, clinical, and tumor characteristics were summarized for the entire study and for both treatment groups in Table 1.

### 3.2. Efficiency

CT or MR imaging follow-up was performed every 3 months after treatment. In the B-TACE group, there was one case that did not receive treatment. During the follow-up, for this particular case, we employed worst-case imputation for sensitivity analysis to evaluate the robustness of the results. The treatment response results of the two groups were summarized in Table 1. The results showed that the 3-month objective response rate (ORR) was significantly higher in the B-TACE group than in the C-TACE group (63.3% [19/30] vs. 10.0% [3/30]; *p* < 0.001). The best overall response at 6 months was found to be significantly higher in B-TACE than in C-TACE (CR: 30.0% [9/30] vs. 3.3% [1/30]; PR: 33.3% [10/30] vs. 6.7% [2/30]). In contrast, disease progression (PD) was significantly higher in the C-TACE group (66.7% [20/30] vs. 20.0% [6/30]). B-TACE also showed a significant advantage in disease control rate (DCR) (80.0% [24/30] vs. 36.7% [11/30]; *p* < 0.001) (Table 2).

**Figure 2:**
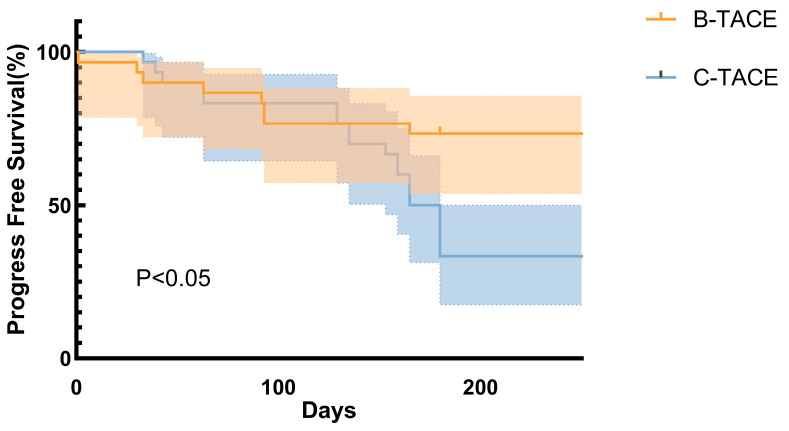
Comparison of Kaplan–Meier survival curves between patients in B-TACE and C-TACE groups (ITT Population).

Survival curves were plotted by Kaplan–Meier method, and Log-Rank test (Mantel-Cox) and Gehan–Breslow–Wilcoxon test were used to compare the survival distribution between B-TACE group and C-TACE group (Figure 2). Log-Rank test results showed that there was a significant difference in survival curves between the two groups (*p* < 0.05). Gehan–Breslow–Wilcoxon test also showed that the difference in survival curves was statistically significant (*p* < 0.05). Median survival was 172.5 days for C-TACE; this was not reached for B-TACE during the follow-up period of this study. Hazard ratio analysis further quantified the risk differences between the two groups. Based on Mantel–Haenszel method, the hazard ratio (HR) for B-TACE group versus C-TACE group was 0.31 (95% CI: 0.148–0.631). The hazard ratio calculated based on the Log-Rank method was 0.30 (95% CI: 0.148–0.608). Both methods yield hazard ratios less than 1, and their 95% confidence intervals did not contain 1.

**Figure 3:**
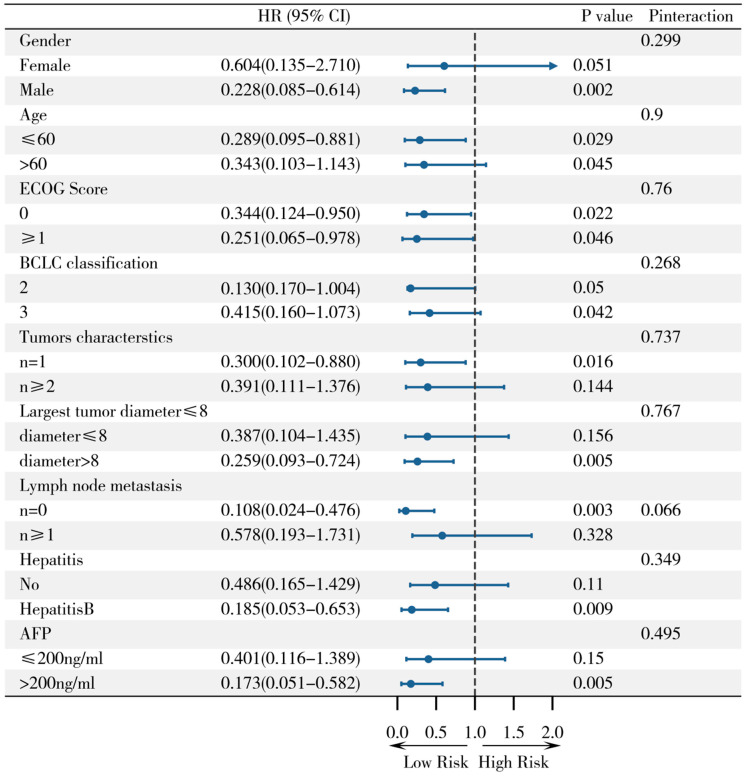
Subgroup analysis of treatment hazard ratio (HR) of B-TACE vs. C-TACE by patient baseline characteristics (ITT population).

To assess the consistency of the survival advantage of B-TACE over C-TACE across patients with different baseline characteristics, we performed subgroup analyses. The results were presented as forest plots (Figure 3). The analysis showed that B-TACE treatment showed a trend towards superiority over C-TACE in most prespecified subgroups. Among them, men (HR = 0.228, 95% CI: 0.085–0.614, *p* = 0.002), ECOG score ≥ 1 (HR = 0.251, 95% CI: 0.065–0.978, *p* = 0.046), maximum tumor diameter >8 cm (HR = 0.259, 95% CI: 0.93–0.724, *p* = 0.005), no lymph node metastasis (HR = 0.108, 95% CI: 0.024–0.476, *p* = 0.003), with hepatitis B (HR = 0.185, 95% CI: 0.93–0.724, *p* = 0.005. The reduction in hazard ratio was statistically significant in the subgroup of patients with Hepatitis B (HR = 0.185, 95% CI: 0.053–0.653, *p* = 0.009) and in the subgroup of patients with AFP *>* 200 ng/mL (HR = 0.173, 95% CI: 0.051–0.582, *p* = 0.005). However, Pinteraction analysis showed that no significant interaction was observed for all baseline characteristics tested (including gender, age, ECOG score, BCLC stage, tumor characteristics, lymph node metastatic status, hepatitis background, and AFP level) (all Pinteraction> 0.05), suggesting that the treatment effect of B-TACE relative to C-TACE was consistent across subgroups, and treatment benefit may not be affected by these baseline characteristics.

**Figure 4:**
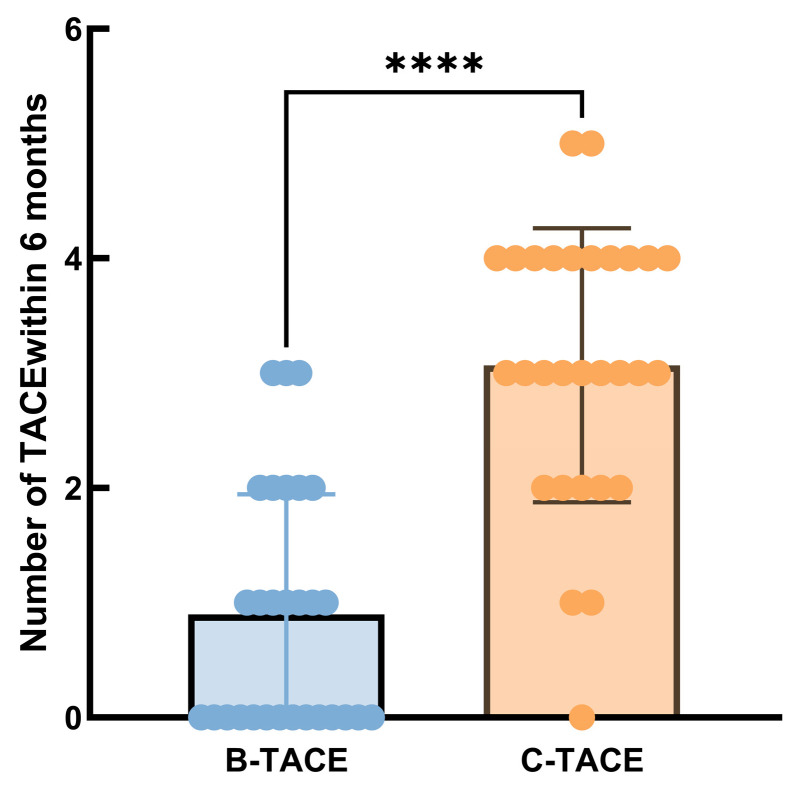
Number of TACE sessions within 6 months post-procedure.The colored dots represent the number of people who have received treatment. Comparison between the B-TACE and C-TACE groups (mITT Population). (**** *p* < 0.0001, Mann-Whitney U test). Central lines represent the median.

During the 6-month follow-up period, there was a significant difference in the postoperative conversion rate between patients in the B-TACE group and those in the C-TACE group. The analysis was performed in the modified intention-to-treat (mITT) population. Ten patients (34.48%, 10/29) in the B-TACE group reached the criteria for radical resection after TACE treatment and underwent successful hepatectomy, while only two patients (6.67%, 2/30) in the C-TACE group completed transformation (*p* = 0.009) (Table 3). The majority of patients with B-TACE achieved resectable status within 3 months after TACE. There was no perioperative death in any of the patients who underwent surgery. There was a significant difference between B-TACE and C-TACE groups in the need for TACE procedures within 6 months after TACE. The median number of procedures in B-TACE group was 1 (*n* = 29) compared with 3 (*n* = 30) in C-TACE group (*p* < 0.001) (Figure 4).

### 3.3. Safety

**Figure 5:**
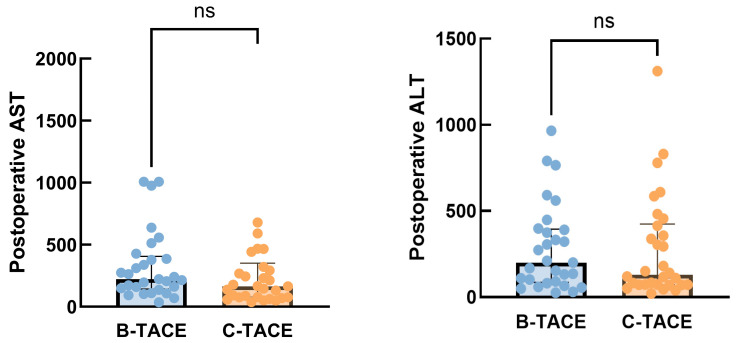
Distribution of postoperative AST levels. Comparison between patients receiving B-TACE and C-TACE. The central line in the box plot represents the median. (*p* = 0.6543, Mann–Whitney U test). Distribution of postoperative ALT levels. Comparison between patients receiving B-TACE and C-TACE. The central line in the box plot represents the median. (*p* = 0.1677, Mann–Whitney U test) (mITT Population). ns means not significant.

AST and ALT levels were compared between B-TACE group and C-TACE group using the Mann–Whitney U test. For AST, the median was 200.0 U/L in B-TACE group (*n* = 29) and 129.5 U/L in C-TACE group (*n* = 30), with no statistical difference between the two groups (Mann–Whitney U = 405, *p* = 0.6543). For ALT, the median was 222.0 U/L in B-TACE (*n* = 29) and 164.5 U/L in C-TACE (*n* = 30), without significant difference (Mann–Whitney U = 343.5, *p* = 0.1677). All analyses were two-tailed tests using exact P values, suggesting that B-TACE and C-TACE did not cause significant differences in postoperative liver function (Figure 5).

The incidence of adverse events was analyzed in the modified ITT population (C-TACE, *n* = 30; B-TACE, *n* = 29), which included all patients who received the allocated treatment. According to CTCAE version 5.0 criteria, the occurrence of postoperative grade ≥ 3 adverse events in 59 patients was systematically evaluated, and the results were summarized in the table below. Table 4 shows that infections (47.5%) and liver function abnormalities (50.8%) are the most common serious adverse events. In the comparison between groups, there is no statistical difference between B-TACE group and C-TACE group in the incidence such as hemoglobin decrease (0% vs. 10.0%, *p* = 0.248), severe liver function abnormality (48.3% vs. 53.3%, *p* = 0.898), high fever (10.3% vs. 30.0%, *p* = 0.121), and significant pain (31.0% vs. 56.7%, *p* = 0.085). Notably, there is a significant difference in the incidence of grade ≥ 3 infections between the two groups (*p* < 0.001). The infection rate of B-TACE group is 17.2% (5/29), significantly lower than that of C-TACE group (76.7% (23/30)). These infection events were defined according to CTCAE 5.0 criteria. Overall, the incidence of Grade ≥ 3 adverse events was low in the B-TACE arm and did not increase the risk of serious liver function injury or hematopoietic toxicity. The results suggested that B-TACE has good safety and tolerability while improving the surgical efficacy and local control rate (Table 4).

### 3.4. BOASP May Predicts Therapeutic Effect

**Figure 6:**
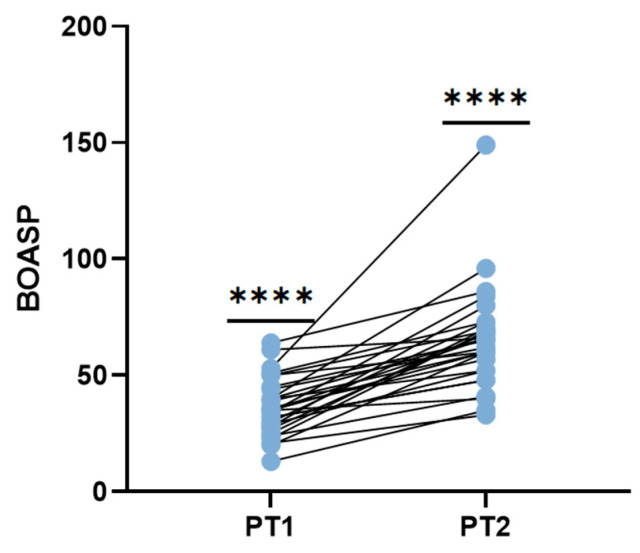
Changes in balloon-occluded arterial stump pressure (BOASP) before and after B-TACE (mITT Population). **** *p* < 0.0001.

Based on the results of the former animal experiments, we further monitored BOASP changes in patients in a prospective clinical study (Figure 6). Intraoperative BOASP increased significantly after embolization (PT2) compared with pre-embolization values (PT1), with a mean difference of 28.69 mmHg (95% CI, 21.36–36.02; *p* < 0.0001, paired *t*-test). This dynamic change was highly consistent with the increasing trend in animal experiments, suggesting that the increase of BOASP is a physiological reflection of embolization effectiveness (Table 5).

**Figure 7:**
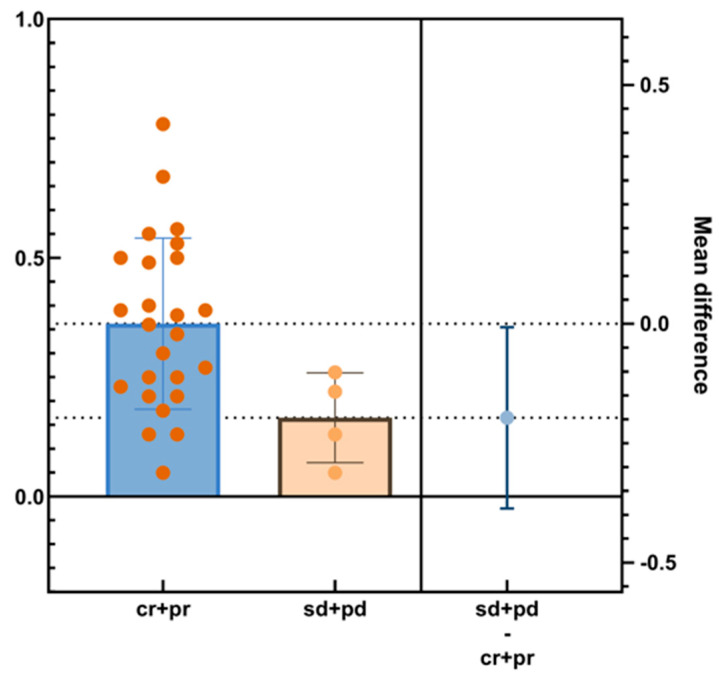
The BOASP of different efficacy subgroups within the B-TACE group at 3 months (mITT Population).

To further explore the relationship between intraoperative BOASP changes and efficacy, we divided patients into response group (CR + PR, *n* = 25) and non-response group (SD + PD, *n* = 4). Unpaired *t*-test results showed significant differences in mean BOASP change between groups (CR + PR: 0.3620 ± 0.092, SD + PD: 0.1650 ± 0.092; mean difference = −0.1970, 95% CI: −0.3868 to −0.0072, t = 2.130, df = 27, *p* = 0.0424). The effect size (eta squared) is 0.1439, suggesting a moderate correlation. However, one-way ANOVA did not show statistically significant differences (F = 3.631, df = 24, 3, *p* = 0.3149). The results suggested that patients with higher BOASP elevation are more likely to achieve a better response (CR + PR), while patients with limited BOASP elevation are more likely to have a poor response (SD + PD) (Figure 7).

**Figure 8:**
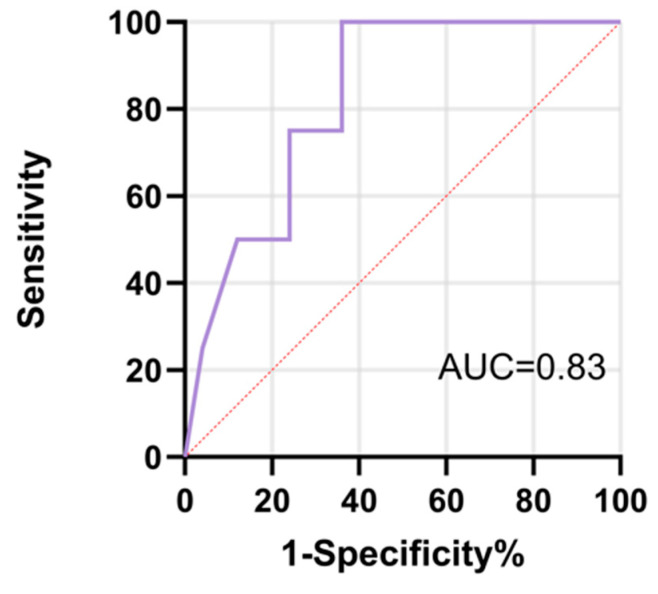
Receiver operating characteristic (ROC) curve of BOASP in preliminary predicting treatment response after B-TACE (mITT Population).

To further assess the clinical significance of BOASP elevation, ROC curve analysis was performed (Figure 8, Table 6). BOASP had a high exploratory value for efficacy (AUC = 0.825, 95% CI: 0.651–0.999, *p* = 0.0398). These results suggested that intraoperative dynamic changes in BOASP not only could reflect hemodynamic changes during embolization but also serve as potential predictors of efficacy (Table 6).

## 4. Discussion

This study highlights the advantages of B-TACE over C-TACE, aligning with the literature’s growing focus on optimizing embolization to address the limitations of conventional methods [14,15,16]. Notably, our findings demonstrate B-TACE’s superiority in treating large HCC (>5 cm), expanding upon previous research that primarily compared B-TACE and C-TACE for 3–5 cm HCC nodules [17]. Despite our study’s advanced disease burden, B-TACE was significantly superior to C-TACE in CR, ORR, PFS, and 6-month re-TACE requirements. Kaplan–Meier survival analysis further revealed that B-TACE significantly prolonged survival, reducing the risk of death by over 70% across multiple prespecified subgroups. These analyses were performed in the modified intention-to-treat (mITT) population, defined as patients who underwent the allocated intervention and had available follow-up data for the corresponding endpoints. These results emphasize B-TACE’s robustness as an effective local treatment for large HCC, particularly in patients where conventional TACE yields suboptimal outcomes [18,19].

To further assess the consistency of efficacy of B-TACE compared to C-TACE across populations with different clinical characteristics, stratified subgroup analyses were performed in this study. The results showed that the survival advantage of B-TACE was maintained in most of the prespecified subgroups and showed a more significant improvement in efficacy in some high-risk populations. Specifically, B-TACE in men (HR = 0.228, 95% CI 0.085–0.614, *p* = 0.002), patients aged >60 years (HR = 0.343, 95% CI 0.103–1.143, *p* = 0.045), ECOG ≥1 (HR = 0.251, 95% CI 0.065–0.978, *p* = 0.046), BCLC stage III (HR = 0.415, 95% CI 0.160–1.073, *p* = 0.042), tumor diameter >8 cm (HR = 0.259, 95% CI 0.093–0.724, *p* = 0.005), absence of lymph node metastasis (HR = 0.108, 95% CI 0.024–0.476, *p* = 0.003), hepatitis B infection (HR = 0.185, 95% CI 0.053–0.653, *p* = 0.009), and AFP > 200 ng/mL (HR = 0.173, 95% CI 0.051–0.582, *p* = 0.005). Interaction analysis showed no significant interaction effects for all clinical characteristics (P_interaction > 0.05), suggesting that the efficacy of B-TACE was consistent and not affected by baseline factors such as age, gender, liver function, or tumor burden. These results suggest that B-TACE not only presents a significant advantage in the overall population but is also particularly suitable for large tumor size, advanced stage, and biologically aggressive patient populations. This finding indicates that B-TACE is not only superior to traditional C-TACE in the overall population but also shows significant therapeutic potential in complex or poor prognosis subgroups, providing a strong clinical basis for individualized intervention strategies.

The 6-month follow-up results revealed that the B-TACE group had a significantly lower proportion of patients experiencing relapse or requiring re-TACE intervention compared to the C-TACE group. This analysis was performed under the modified ITT principle, not included due to unavailable data cases with missing follow-up data. This finding suggests that B-TACE provides more durable local control. The improved efficacy may be due to the complete occlusion of tumor microcirculation and a more uniform distribution of chemotherapy drugs under balloon occlusion, which reduces residual blood supply and the risk of recurrence. The postoperative conversion rate in B-TACE group was 34.5% (10/29), significantly higher than 6.7% (2/30, *p* = 0.009) in C-TACE group. This indicates that B-TACE can achieve stage reduction on the basis of local control, creating an opportunity for secondary radical resection for some patients in the middle and late stages.

Although balloon embolization occlusive procedure (B-TACE) can enhance the intensity of blood flow occlusion, in the analysis of adverse events reported in Table 4 were CTCAE (version 5.0) grade ≥3. The incidence of severe infection was significantly higher in the C-TACE group, while no significant differences were observed for other severe adverse events.

Overall, infections (47.5%) and abnormal hepatic function (50.8%) were the most common SAEs. There was no significant difference between B-TACE and C-TACE in grade ≥3 events of hemoglobin decrease, abnormal liver function, fever, and pain (all *p* > 0.05), but the incidence of infection was significantly lower in B-TACE group (17.2% vs. 76.7%, *p* < 0.001), possibly due to more precise perfusion control and reduced retrograde flow 20. No device-related complications associated with balloon manipulation were observed. This result suggests that B-TACE does not increase the risk of postoperative complications while enhancing blood flow occlusion but rather reduces infection rates by reducing non-targeted reflux and tissue contamination of chemotherapeutic agents. This safety result provides a good risk-benefit basis for the broad clinical use of B-TACE.

The core innovation of this study is that intraoperative BOASP monitoring may serve as an indicator of embolic adequacy and predictor of treatment response. Animal experiments have shown that embolization causes repeatable increases in arterial pressure and that a sustained curve of increase is associated with greater vascular occlusion. The initial variability observed between different univariate analytical approaches highlights the importance of selecting statistical methods appropriate for paired measurements. Accordingly, paired analyses were used to more accurately assess within-patient changes in BOASP. These mechanistic insights were translated into clinical settings, with patients receiving B-TACE exhibiting significant increases in BOASP after embolization. More importantly, the magnitude of BOASP elevation correlated with treatment outcome: responders (CR + PR) had significantly higher BOASP elevation than nonresponders (SD + PD). ROC curve analysis further confirmed BOASP’s potential predictive power with an AUC of 0.825, underscoring its potential as a hemodynamic indicator of treatment efficacy. Unlike traditional imaging-based assessments performed only after treatment, intraoperative BOASP measurements are instantaneous and objective. First, BOASP monitoring provides real-time feedback during B-TACE, allowing the operator to objectively assess embolic adequacy and determine whether additional embolic material is needed. Second, BOASP’s predictive power helps identify potential nonresponders intraoperatively, allowing timely adjustment of treatment strategies, including combination or switching to systemic therapy. Third, the integration of BOASP monitoring into B-TACE standardized processes is expected to reduce operational inconsistencies due to differences in operator experience and improve repeatability and standardization of treatment.

The mechanism behind these observations is that balloon occlusion increases intraarterial pressure, reduces arterial inflow, and promotes deeper penetration and longer retention of chemotherapy drugs within tumor microvessels [17,20]. This hemodynamic modulation may explain the superior tumor control and survival rates of B-TACE over C-TACE. In addition, BOASP elevation provides a direct physiological reading of embolic adequacy, complementing a potentially subjective angiographic impression. From a pathophysiological perspective, BOASP reflects the hemodynamic response of the tumor-feeding artery under balloon occlusion. An increase in BOASP indicates effective interruption of antegrade arterial flow, redistribution of perfusion pressure, and increased resistance within the embolized vascular bed. These changes may facilitate enhanced retention of chemotherapeutic agents and embolic materials within the tumor microvasculature. Accordingly, BOASP should be regarded as a dynamic intraoperative hemodynamic indicator of embolization adequacy rather than a definitive biological biomarker. Therefore, integration of BOASP monitoring into routine B-TACE procedures may enhance standardization of procedures and enable personalized adjustment of embolic endpoints.

Despite these encouraging findings, certain limitations should be acknowledged. First, this is a single-center study with a small sample size, which may limit the statistical ability to detect subgroup interactions. And although baseline characteristics were generally balanced, the higher prevalence of elevated AFP in the B-TACE group may have introduced residual confounding; although, the survival benefit observed after stratified analysis remained strong. Second, although intraoperative BOASP in B-TACE demonstrated potential utility as an exploratory indicator of treatment response, further validation in larger cohorts is required, and the cut-off threshold for clinical decision making and the measurement of optimal range of change in intraoperative BOASP for B-TACE need to be further validated in a larger, multicenter prospective randomized controlled study. Finally, the study focused on short-and medium-term outcomes, and whether intraoperative BOASP predicts long-term survival or recurrence patterns merits further longitudinal investigation.

## 5. Conclusions

This prospective study compared balloon-occluded transarterial chemoembolization (B-TACE) with conventional TACE (C-TACE) in patients with large hepatocellular carcinoma and explored the potential role of intraoperative balloon-occluded arterial stump pressure (BOASP) as a hemodynamic indicator of embolization adequacy based on preclinical and clinical observations. The results suggest that B-TACE was associated with improved treatment response and survival outcomes compared with C-TACE. The therapeutic effect appeared generally consistent across prespecified subgroups and may be more pronounced in patients with higher-risk disease profiles. In addition, Exploratory ROC analyses indicated a potential discriminative ability of BOASP for treatment efficacy, while B-TACE was also associated with acceptable safety and a higher rate of successful surgical conversion. Taken together, these findings support the feasibility and potential clinical benefit of B-TACE in the management of advanced hepatocellular carcinoma and highlight the value of incorporating intraoperative hemodynamic information into interventional decision-making. However, given the exploratory nature of the BOASP analyses and the limited sample size, these observations should be interpreted with caution. Further validation in larger, multi-center randomized controlled trials is warranted to define optimal BOASP thresholds and to clarify its role in individualized TACE strategies across different HCC subtypes.

## Figures and Tables

**Table 1:** Demographic baseline characteristics of enrolled patients (ITT Population).

	Total (*n* = 60)	B-TACE (*n* = 30)	C-TACE (*n* = 30)	*p*-Value
Gender				1.00
Female	11 (18.3%)	6 (20.0%)	5 (16.7%)	
Male	49 (81.7%)	24 (80.0%)	25 (83.3%)	
Age (Median [Q1, Q3])	59.1 ± 12.3	61.0 (50.0, 71.5)	56.5 (47.8, 65.8)	0.07
ECOG.Score, NO (%)				0.56
0	38 (63.3%)	18 (60.0%)	20 (66.7%)	
1	21 (35.0%)	11 (36.7%)	10 (33.3%)	
2	1 (1.7%)	1 (3.3%)	0 (0%)	
Child-Pugh, NO (%)				0.42
a	53 (88.3%)	28 (93.3%)	25 (83.3%)	
b	7 (11.7%)	2 (6.7%)	5 (16.7%)	
Cirrhosis, NO (%)				0.17
0	40 (66.7%)	23 (76.7%)	17 (56.7%)	
1	20 (33.3%)	7 (23.3%)	13 (43.3%)	
Portal.Vein.Tethering, NO (%)	15 (25.0%)	5 (16.7%)	10 (33.3%)	0.23
Portal.Hypertension, NO (%)	7 (11.7%)	2 (6.7%)	5 (16.7%)	0.42
Pathological. NO (%)				0.29
Hcc	55 (91.7%)	29 (96.7%)	26 (86.7%)	
Hcc-Icc	5 (8.3%)	1 (3.3%)	4 (13.3%)	
BCLC.classification, NO, (%)				0.07
a	1 (1.7%)	1 (3.3%)	0 (0%)	
b	22 (36.7%)	7 (23.3%)	15 (50.0%)	
c	37 (61.7%)	22 (73.3%)	15 (50.0%)	
Tumors.characterstics, NO, (%)				0.07
Solitary	36 (60.0%)	22 (73.3%)	14 (46.7%)	
Multi focal	24 (40.0%)	8 (26.7%)	16 (53.3%)	
Largest.tumor.diameter (Median [Q1,Q3]), cm	9.6 ± 3.7	9.1 (6.4, 13.3)	8.8 (6.8, 12.0)	0.76
Lymph.node.metastasis, NO, (%)	27 (45.0%)	14 (46.7%)	13 (43.3%)	1.00
Hyperemia, NO (%)	17 (28.3%)	8 (26.7%)	9 (30.0%)	1.00
Diabetes.Mellitus, NO (%)	10 (16.7%)	6 (20.0%)	4 (13.3%)	0.86
Hepatitis.B, NO (%)	34 (56.7%)	17 (56.7%)	17 (56.7%)	1.00
AFP, NO, (%)				<0.01
≤200 ng/mL	31 (51.7%)	10 (33.3%)	21 (70.0%)	
>200 ng/mL	29 (48.3%)	20 (66.7%)	9 (30.0%)	
PIVKAIVKA-II, NO (%)				1.00
≥2000 ng/mL	22 (36.7%)	11 (36.7%)	11 (36.7%)	
<2000 ng/mL	38 (63.3%)	19 (63.3%)	19 (63.3%)	

HCC, hepatocellular carcinoma; ECOG, Eastern Cooperative Oncology Group performance status; Child-Pugh, Child-Turcotte-PughCTP; BCLC, Barcelona Clinic Liver Cancer; Portal.Vein.Tethering, Portal Vein Tethering Sign; AFP, alpha-fetoprotein; PIVKAIVKA-II, Protein Induced by Vitamin K Absence or Antagonist-II.

**Table 2:** The therapeutic effect evaluation results of B-TACE and C-TACE patients (ITT Population).

	Total (*n* = 60)	B-TACE (*n* = 30)	C-TACE (*n* = 30)	*p*-Value
Objective response (CR + PR)	30 (50.0%)	25 (83.3%)	5 (16.7%)	<0.001
6 months Best overall response, *n* (%)				<0.001
CR	10 (16.7%)	9 (30.0%)	1 (3.3%)	
PR	12 (20.0%)	10 (33.3%)	2 (6.7%)	
SD	12 (20.0%)	5 (16.7%)	7 (23.3%)	
PD	26 (43.3%)	6 (20.0%)	20 (66.7%)	
DCR (CR + PR + SD), n (%)	35 (58.3%)	24 (80.0%)	11 (36.7%)	<0.001

Note: ORR (objective response rate) is defined as CR + PR, and the measurement time is 3 months after operation; DCR (disease control rate) is defined as CR + PR + SD; χ^2^ test is used for statistical method.

**Table 3:** Comparison of surgical conversion between B-TACE and C-TACE groups. Data are presented as number (percentage). Statistical comparison was performed using the χ^2^ test. *p* < 0.05 was considered statistically significant. Analyses were performed in the modified intention-to-treat (mITT) population, defined as patients who underwent the allocated intervention and had available data for the corresponding endpoints.

	Total (*n* = 59)	B-TACE (*n* = 29)	C-TACE (*n* = 30)	*p*
Surgery	21	10 (34.48%)	2 (6.67%)	0.009

**Table 4:** Adverse events (CTCAE grade ≥ 3) in the B-TACE and C-TACE groups, and the comparison between groups is performed by χ^2^ test. *p* < 0.05 is considered statistically significant. Adverse events were graded according to CTCAE version 5.0.

	Total (*n* = 59)	B-TACE (*n* = 29)	C-TACE (*n* = 30)	*p*-Value
Hb < 80	3 (5.1%)	0 (0%)	3 (10.0%)	0.248
Hepatic function abnormality	30 (50.8%)	14 (48.3%)	16 (53.3%)	0.898
Infection	28 (47.5%)	5 (17.2%)	23 (76.7%)	<0.001
Fever	12 (20.3%)	3 (10.3%)	9 (30.0%)	0.121
Pain	26 (44.1%)	9 (31.0%)	17 (56.7%)	0.085

Hb, Hemoglobin; Hepatic function abnormality, AST (Aspartate Aminotransferase)/ALT (Alanine Aminotransferase) > 5.0 × ULN (Upper Limit of Normal).

**Table 5:** Single-sample *t*-test of BOASP levels before and after B-TACE, BOASP significantly increased after B-TACE compared with baseline (PT1 vs. PT2, *p* < 0.0001).

Comparison (Paired)	Sample Size (n)	BOASP (Mean ± SD, mmHg)		Paired *t*-Test Results			
		PT1	PT2	Mean Difference (mmHg)	t (df)	95% CI of Difference	*p* Value
PT1 vs. PT2	29	36.41 ± 12.28	65.10 ± 21.92	28.69	8.014 (28)	21.36–36.02	<0.0001

**Table 6:** ROC analysis of BOASP in preliminary predicting treatment response.

Variable	AUC	SE	95% CI	*p* Value
BOASP	0.825	0.0886	0.651–0.999	0.0398

## Data Availability

The original contributions presented in this study are included in the article/Supplementary Material. Further inquiries can be directed to the corresponding authors.

## Supplementary Materials

The following supporting information can be downloaded at https://www.mdpi.com/article/10.3390/jcm15020668/s1, Table S1. CONSORT 2010 Checklist of Information to Include When Reporting a Randomised Trial.

## Appendix A. Microballoon Catheter Characteristics

In order to reduce experimental errors and ensure the accuracy of experimental results, we uniformly used a type of 2.7 fr tip dual-lumen compliant microballoon catheter: Occlur (Huanxin Medical Clinical Products Company, Suzhou, China). Details about Occlur are summarized in Figure 2.
